# Letter from the Editor in Chief

**DOI:** 10.19102/icrm.2024.15084

**Published:** 2024-08-15

**Authors:** Devi Nair


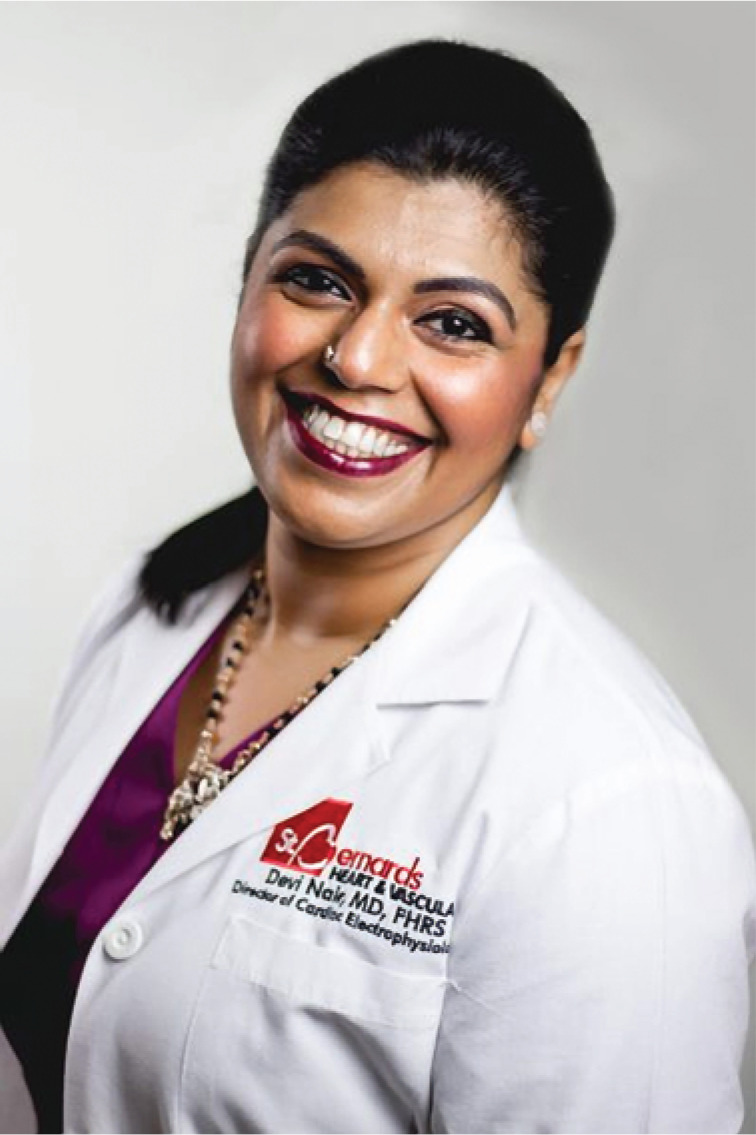


Dear readers,

Welcome to the August 2024 issue of *The Journal of Innovations in Cardiac Rhythm Management*. This month, we are excited to feature a comprehensive and insightful paper by Pavani et al. titled “Comparative Analysis of Clinical Outcomes of High-power, Short-duration Ablation versus Low-power, Long-duration Ablation Strategy in Patients with Atrial Fibrillation: A Comprehensive Umbrella Review of Meta-analyses.”^1^

Atrial fibrillation (AF) continues to be a major clinical challenge, affecting millions worldwide and contributing to significant morbidity and healthcare costs. The quest to optimize catheter ablation techniques to improve patient outcomes is ongoing, and this month’s featured study by Pavani et al. is a testament to the advancements in this field. Their umbrella review meticulously evaluates the safety, efficacy, and potential of high-power, short-duration (HPSD) ablation as an alternative to the traditional low-power, long-duration (LPLD) approach. By integrating data from multiple systematic reviews and meta-analyses, the authors provide robust evidence supporting the superiority of HPSD ablation in reducing AF recurrence and esophageal thermal injury, while also highlighting its efficiency in procedural aspects.

Pavani et al.’s research offers a rigorous comparative analysis that considers data from 11 studies, integrating findings from 6 randomized controlled trials and 26 observational studies. The study’s primary outcomes demonstrate that the HPSD approach significantly reduces AF recurrence and the risk of esophageal thermal injury compared to the LPLD strategy. Moreover, secondary outcomes indicate improved procedural efficiency with HPSD, including reduced total procedure, fluoroscopy, and ablation times. These findings are pivotal, as they suggest that the HPSD method not only enhances clinical efficacy but also optimizes operational workflow, potentially leading to better patient experiences and outcomes.

To further enrich our understanding, we are also pleased to include in this issue an expert commentary by Drs. Petrovic and Kantharia,^2^ who provide a deep dive into the clinical implications of the findings reported by Pavani et al., emphasizing the critical importance of achieving transmural and contiguous lesions for successful pulmonary vein isolation. Their commentary elucidates how the balance between resistive and conductive heating in the HPSD approach enhances lesion quality while minimizing collateral damage. Petrovic and Kantharia also address the challenges and limitations noted in the aforementioned study, such as the heterogeneity of primary data and the absence of a uniform definition for high power.

Importantly, the commentary by Petrovic and Kantharia also reaches beyond the immediate findings of the work by Pavani et al. to explore the evolving landscape of AF ablation technologies. They discuss emerging techniques, such as very-high-power ablation and non-thermal pulsed-field ablation (PFA). The introduction of the QDOT MICRO™ catheter (Biosense Webster, Diamond Bar, CA, USA), capable of delivering 90 W for very short durations, and the regulatory approval of PFA technology represent significant milestones in the field. These innovations promise to further refine the balance between efficacy and safety in AF ablation, potentially transforming standard practice.

The synergy between Pavani et al.’s comprehensive review and the expert insights shared by Petrovic and Kantharia provides a holistic view of the current landscape and future prospects of AF ablation techniques. Their contributions highlight the dynamic nature of electrophysiology and the continuous efforts to refine and enhance therapeutic strategies for better patient outcomes. The insights gained from this month’s issue of the journal not only advance our understanding but also inspire further research and innovation in the field.

We hope that this issue of *The Journal of Innovations in Cardiac Rhythm Management* will serve as a valuable resource for clinicians, researchers, and all those dedicated to advancing the field of cardiac rhythm management. As always, we are grateful for your continued support and readership.

Sincerely,


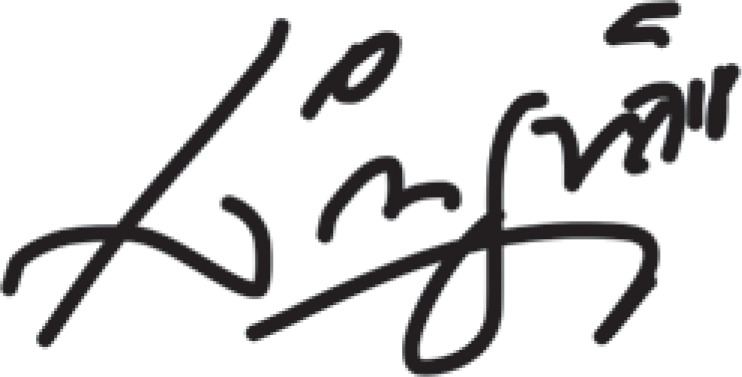


Dr. Devi Nair, md, facc, fhrs

Editor-in-Chief

*The Journal of Innovations in Cardiac Rhythm Management*

Director of the Cardiac Electrophysiology & Research,

St. Bernard’s Heart & Vascular Center, Jonesboro, AR, USA

White River Medical Center, Batesville, AR, USA

President/CEO, Arrhythmia Research Group

Clinical Adjunct Professor, University of Arkansas for Medical Sciences

Governor, Arkansas Chapter of American College of Cardiology

drdgnair@gmail.com
